# Hypoxia mitigation by manganese-doped carbon dots for synergistic photodynamic therapy of oral squamous cell carcinoma

**DOI:** 10.3389/fbioe.2023.1153196

**Published:** 2023-04-20

**Authors:** Zhe Zhang, Yongzhi Xu, Tingting Zhu, Zhiqin Sang, Xiaoli Guo, Yu Sun, Yuanping Hao, Wanchun Wang

**Affiliations:** ^1^ School of Stomatology of Qingdao University, Qingdao, China; ^2^ Qingdao Stomatological Hospital Affiliated to Qingdao University, Qingdao, China

**Keywords:** acidic H_2_O_2_-driven oxygenator, manganese-doped carbon dots, photodynamic therapy, oral squamous cell carcinoma, tumor microenvironment

## Abstract

Photodynamic therapy (PDT) is widely used for cancer treatment due to its non-invasive and precise effectiveness, however, hypoxia in the tumor microenvironment greatly limits the efficacy of photodynamic therapy. Compared with conventional photosensitizers, carbon dots (CDs) have great potential. Therefore, developing a water-soluble, low-toxicity photosensitizer based on CDs is particularly important, especially one that can enhance the photodynamic efficacy using the tumor microenvironment to produce oxygen. Herein, manganese-doped carbon dot (Mn-CDs, ∼2.7 nm) nanoenzymes with excellent biocompatibility were prepared by a solvothermal method using ethylenediaminetetraacetic acid manganese disodium salt hydrate and o-phenylenediamine as precursors. TEM, AFM, HR-TEM, XRD, XPS, FT-IR, ζ potential, DLS, UV-Vis, and PL spectra were used to characterize the Mn-CDs. Cancer resistance was assessed using the CCK-8 kit, calcein AM versus propidium iodide (PI) kit, and the Annexin V-FITC/PI cell apoptosis assay kit. The obtained Mn-CDs have excellent near-infrared emission properties, stability, and efficient ^1^O_2_ generation. Notably, the manganese doping renders CDs with catalase (CAT)-like activity, which leads to the decomposition of acidic H_2_O_2_ *in situ* to generate O_2_, enhancing the PDT efficacy against OSCC-9 cells under 635 nm (300 mW·cm^−2^) irradiation. Thus, this work provides a simple and feasible method for the development of water-soluble photosensitizers with oxygen production, presenting good biosafety for PDT in hypoxic tumors.

## 1 Introduction

Oral cancer is a malignant tumor that occurs in the oral cavity and oropharynx, of which oral squamous cell carcinoma (OSCC) is the most common, accounting for more than 90% of oral cancers (Wong and Wiesenfeld, 2018; Inchingolo et al., 2020). Modern cancer treatment aims to improve disease-free progression-free survival, control the extent of local lesions and reduce disease recurrence. Therefore, emerging therapeutic treatments, such as targeted biologic therapy (Liu, 2019), lytic virus therapy (Gujar et al., 2019), gene therapy (gene editing and silencing) (Bryan Bell, 2021), nanotherapeutics (Zheng et al., 2021), photothermal and photodynamic therapies (Algorri et al., 2021), etc., are gaining attention and achieving promising results.

Photodynamic therapy (PDT), which uses photosensitizers (PSs) to convert surrounding oxygen molecules into cytotoxic reactive oxygen species (ROS) under specific laser irradiation has been a minimally invasive treatment with high specificity, negligible drug resistance and few side effects that have been rapidly developed in the past decade (Yang et al., 2018). However, oxygen dependence limits its application within solid tumors (Liu et al., 2022; Zhang et al., 2022). Hypoxia is an important feature of the tumor microenvironment, usually caused by tumor cell proliferation and abnormal angiogenesis, which would limit the production of ROS and greatly reduce the efficacy of PDT (Anderson and Simon, 2020; Wei et al., 2021). Also, hypoxia can promote tumor metastasis and resistance to various treatments. In addition, the rate of H_2_O_2_ production is increased in cancer cells compared to normal cells (up to 0.5 nmol/104 cells/h), resulting in higher H_2_O_2_ levels in tumors than in normal tissues (Yang et al., 2020). Importantly, as a typical feature of tumour microenvironment (TME), overexpressed H_2_O_2_ is used to trigger responsive drug release in chemotherapy or to generate endogenous O2 used to supplement O2 required for PDT treatment (Zheng et al., 2016). For this purpose, oxygen generation based on the catalytic decomposition of H_2_O_2_ by inorganic nanoenzymes (nanomaterials with enzymatic activity) has been reported (Gao et al., 2019), such as cerium (Ce) (Wang et al., 2020), gold (Au) (Kong et al., 2020), platinum (Pt) (Li et al., 2020) and copper (Cu) (Sun et al., 2020). For example, MnO_2_ nanoparticles and MnO_2_-containing nanoparticles, which are widely studied, can react with H_2_O_2_ and H^+^ in TME to generate large amounts of oxygen *in situ* to overcome the tumor hypoxic environment and improve the efficacy of PDT, and change the pH by redox reaction with acidic H_2_O_2_ in TME (Tao et al., 2022). However, the current construction of nanodrug systems for PDT therapy based on manganese dioxide suffer from the following limitations: 1) the preparation process is complicated and cumbersome; 2) the drug or PSs needs to be loaded on the MnO2 nanoparticles (Liu et al., 2022). Therefore, there is still a great need to establish a simple method to prepare manganese-based nanodrug.

**SCHEME 1:**
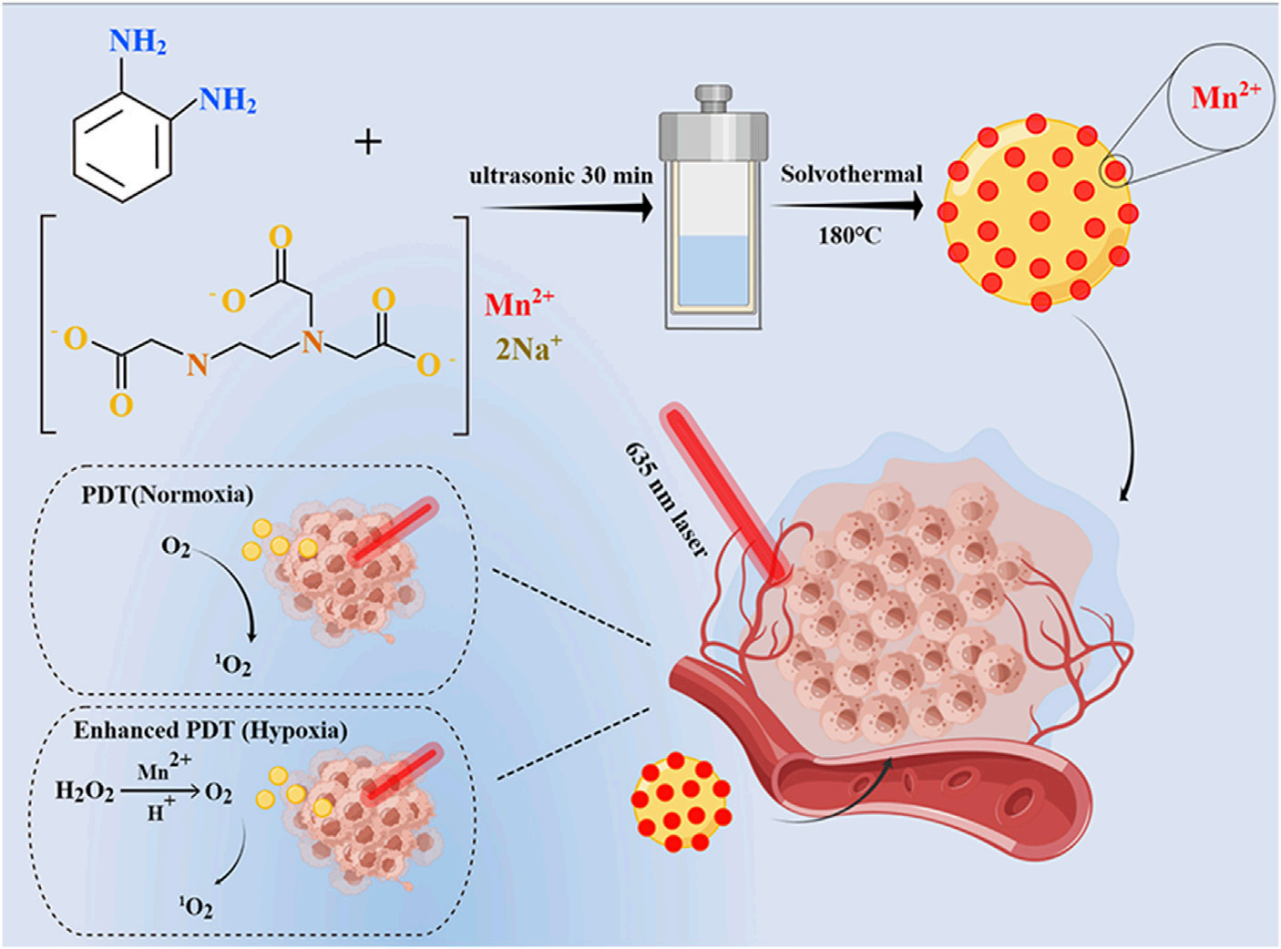
Schematic illustration of Mn-CDs as catalase-like to improve the anticancer efficiency of PDT.

Carbon dots (CDs) have been widely noticed in cancer therapy due to their superior biocompatibility, stable fluorescence (FL) emission, easy surface functionalization, and more importantly, potential light-mediated therapeutic functions such as PDT (Hu et al., 2021; Lv et al., 2021) and photothermal therapy (PTT) (Jia et al., 2018a; Wang et al., 2019). Wang et al. have prepared an acidic pH/high H_2_O_2_-responsive Mn-CDs (diameter of ≈4.2 nm) using manganese II) phthalocyanine (Mn-Pc) as a raw material (Jia et al., 2018a). The Mn-CDs have similar properties to MnO_2_ as a nano-photosensitizer for *in situ* oxygen generation to improve the efficacy of PDT in hypoxic tumors (Jia et al., 2018a; Irmania et al., 2020). However, such hydrophobic Mn-CDs need to self-assemble with DSPE-PEG to enhance biocompatibility and water solubility. To address this limitation, in this research, we first synthesized water-soluble Mn-CDs proceeded from ethylenediaminetetraacetic acid manganese disodium salt hydrate and o-phenylenediamine via a simple one-pot solvothermal method (Scheme 1). The obtained Mn-CDs possess excellent near-infrared emission properties, stability, and efficient 1O_2_ generation. Notably, *in vitro* studies have demonstrated that these fluorescent Mn-CDs were able to decompose acidic H_2_O_2_ *in situ* to produce O_2_, thus efficiently enhancing the PDT efficacy.

## 2 Materials and methods

### 2.1 Materials

Ethylenediaminetetraacetic acid manganese disodium salt hydrate (NaMnEDTA), o-phenylenediamine (OPD), methylene blue (MB) and ethylenediaminetetraacetic acid tetrasodium salt hydrate (EDTANa) were purchased from Aladdin Chemistry Co., Ltd. (Shanghai, China). Dialysis bag (cutoff Mn: 100–500 Da) was procured from Yuanye Biotechnology Co., Ltd. (Shanghai, China). 1,3-Diphenylisobenzofuran (DPBF) was obtained from Macklin Regent (Shanghai, China). Anhydrous ethanol was provided from Sinopharm Chemical Reagent Co., Ltd. (Shanghai, China). Counting Kit-8 (CCK-8) was purchased from Solarbio Science & Technology Co., Ltd. (Beijing, China). Singlet Oxygen Sensor Green reagent (SOSG) was provided from MesGen Biotechnology. (Shanghai, China). Procell Life Science & Technology Co., Ltd. (Wuhan, China) was our provider for Dulbecco’s modified eagle medium (DMEM), fetal bovine serum (FBS), 0.25% trypsin solution, phosphate buffered solution (PBS, 0.01 M, pH 7.4) and Annexin V-FITC/PI Apoptosis Detection Kit. Calcein AM and Propidium iodide (PI) were obtained from Meilunbio Co., Ltd. (Dalian, China). Penicillin/streptomycin was purchased from Biological Industries (Israel). All chemicals were used as received without further purification.

### 2.2 Cell culture and animals

Oral squamous cell carcinoma (OSCC)-9 cells were purchased from China Center for Type Culture Collection (CCTCC) and cultured in DMEM containing 10% (v/v) FBS and 1% (v/v) penicillin-streptomycin. Cells were cultured at 37°C in a humidified incubator under 5% CO2.

Sprague Dawley (SD) rats (male, ∼6 weeks old) were purchased from Jinan Pengyue Experimental Animal Breeding Co., Ltd. and maintained in Qingdao University’s Laboratory Animal Center in a specific pathogen-free (SPF) environment. All procedures performed in the study were in accordance with the Ethics Committee of Qingdao Stomatological Hospital Affiliated of Qingdao University Certificate (contract grant 2021KQYX032) and with the National Research Council’s Guide for the Care and Use of Laboratory Animals ethical standards.

### 2.3 Preparation and purification of Mn-CDs and pristine CDs

To synthesize water-soluble Mn-CDs for *in situ* oxygen production to improve the efficacy of photodynamic therapy, we employed NaMnEDTA and OPD as raw materials for the first time to synthesize Mn-CDs via one-pot hydrothermal method. The synthetic process is detailed in Scheme 1. Mn-CDs were synthesized with a solvothermal method. NaMnEDTA (280 mg) and o-phenylenediamine (95 mg) were added into 30 mL hydrochloric acid solution (0.6 M). After 30 min of sonication, the mixture was rapidly sealed in a Teflon-lined stainless steel autoclave and heated at 180°C for 6 h. Then the deep blue solution was naturally cooled down to room temperature and dispersed by ultrasound for 1 h. The obtained mixture was passed through the 0.22 μm filter membrane to acquire a transparent solution. Then the obtaining products were dialyzed in a dialysis bag in deionized water for 24 h. Finally, the purified Mn-CDs were lyophilized in a freeze-vacuum dryer (SCIENTZ-10N, XinZhi freeze-drying equipment Co., Ltd. Ningbo, China) and stored in a refrigerator at 4°C. The Mn-free CDs were obtained via the same synthesis procedure as above using EDTANa.

### 2.4 Physico-chemical characterization

i) The morphology and size of Mn-CDs and CDs were characterized using Transmission electron microscopy (TEM) at an acceleration voltage of 100 kV (JEM-2100UHR, JEOL, Japan). The lattice spacing of Mn-CDs and CDs was characterized using High-resolution transmission electron microscopy (HR-TEM) at an acceleration voltage of 200 kV (JEM-2100F, Japan).
ii) The height of Mn-CDs and CDs was measured by Atomic force microscopy (AFM) (Dimension Icon, Bruker, United States) using a scan step of 2 MHz.
iii) The surface charge of nanoparticles was detected with Zetasizer Nano ZS (Malvern instrument, United Kingdom) at 25°C.
iv) The surface chemical elements and chemical bonds of Mn-CDs and CDs were analyzed by X-ray photoelectron spectroscopy (XPS) operating with step size 20.0 eV and scan number 5 (ESCALAB Xi, Thermo Fisher, United States).
v) The crystal structure of Mn-CDs and CDs was examined by X-ray diffraction (XRD, MiniFlex 600, Rugaku Corporation, Japan) under Cu Kα radiation with voltage of 40 kV and current of 40 mA.
vi) The functional groups of Mn-CDs and CDs were characterized using a Nicolet iN10 Fourier transform infrared (FT-IR) spectrometer (Thermo Fisher Scientific, Waltham, MA, United States) in the range of 500–4,000 cm^−1^ with a scan resolution of 2 cm^−1^ during 32 scans.
vii) The Ultraviolet-visible (UV–Vis) spectra of Mn-CDs and CDs was recorded on a UV–8000 spectrophotometer (Shanghai Metash Instruments, China).
viii) The excitation and emission spectra of Mn-CDs and CDs were detected using a fluorescence spectrophotometer (Edinburgh Instrument FS5, United Kingdom). The data interval and scanning speed were 1.0 nm and 2000 nm/min, respectively.
ix) For stability experiments, Mn-CDs or CDs were dispersed in different media solutions (including water, PBS, DMEM and FBS) at room temperature for 2 weeks to observe the dissolution and dispersion. Meanwhile, the hydrated particle size of Mn-CDs in the above media solutions was measured by Zetasizer Nano ZS (Malvern instrument, United Kingdom) at 25°C. In addition, the excitation and emission spectra of Mn-CDs in the above media were detected using a fluorescence spectrophotometer (Edinburgh Instrument FS5, United Kingdom). The data interval and scanning speed were 1.0 nm and 2000 nm/min, respectively.
x) The photodynamic efficiency of Mn-CDs and CDs in extracellular:

The dissolved oxygen concentration of Mn-CDs in different reaction systems was determined using a JPBJ-610L portable dissolved oxygen meter (JPBJ-610L, Rex Electric Chemical, Shanghai Oshitol Industrial Co., Ltd. Shanghai, China).

DPBF was used as a reactive oxygen trapper to detect the photodynamic effect of Mn-CDs (Algorri et al., 2021). DPBF solution (2 mg/mL, dissolved in anhydrous ethanol) was added to Mn-CDs (20 μg/mL, 3 mL) solution in the absence or presence of H_2_O_2_ (50 μM, pH = 6.5, dissolved in deionized water), and the absorbance of the mixture at 412 nm was recorded on a UV–Vis spectrophotometer after irradiation with a 635 nm laser for 5 min. Detection of 1O2 production by CDs using the same method as for Mn-CDs.

To calculate the 1O2 quantum yield, we used the same method as for Mn-CDs to detect the 1O2 generated by MB. The first-order exponential fit of the obtained curves was performed with Origin 2019b software to calculate the decay time of the absorption intensity at 412 nm with irradiation time. According to the previously reported method (Jia et al., 2018b), using the MB solution as a standard, the release of Mn-CDs to 1O2 was calculated based on the relationship of Eq. 1:

$$\emptyset_{Mn-CDs}=\emptyset_{MB}\;\frac{t_{MB}}{t_{Mn-CDs}}$$ (1)

where tMn-CDs and tMB correspond to the time at which the decrease in DPBF absorption is adjusted to first-order exponential decay in the presence of Mn-CDs and MB, respectively, and ΦMB is the 1O2 quantum yield of free MB in ethanol solution, noted as 0.49.

### 2.5 Biological evaluation

#### 2.5.1 Photodynamic performance of the Mn-CDs and CDs in the simulated tumor microenvironment

Detection of intracellular 1O2 production by Mn-CDs employing SOSG reagent. First, OSCC-9 cells (1 × 105 per well) were inoculated into confocal culture plates until adherence. The old medium was discarded, and 500 μL of Mn-CDs solution (100 μg/mL) was added to continue the incubation for 4 h. Next, cells were fixed with 4% paraformaldehyde for 20 min and then incubated with SOSG (1 mg/mL, 5 μL) for 15 min. After the addition of H_2_O_2_ (pH = 6.5), the cells were irradiated with a 635 nm laser (300 mW·cm-2) for 5 min and imaged with an SP8 Laser scanning confocal microscopy (LSCM, Leica Microsystems). Subgroups without the addition of hydrogen peroxide served as control.

#### 2.5.2 Cellular uptake efficacy

OSCC-9 cells (2.5 × 104 per well) were inoculated in confocal plates and cultured for 24 h. After treatment with 500 μL of DMEM containing Mn-CDs (100 μg/mL) for 4 h, the cells were washed with PBS (0.01M, pH 7.4) to remove non-specifically bond Mn-CDs before observing with an SP8 LSCM at an excitation wavelength of 610 nm.

#### 2.5.3 Photodynamic therapy of the Mn-CDs and CDs in the simulated tumor microenvironment

Validation of the biosafety of Mn-CDs and CDs using the CCK-8 assay. OSCC-9 cells (5 × 103 per well) were incubated in 96-well plates in 5% CO2 at 37°C for 24 h. The co-culture was continued by adding different concentrations of Mn-CDs or CDs solution (0, 25, 50, 100, 200, 300 μg/mL) for 24 h. Then, the old medium was replaced by CCK-8 solution, and the absorbance at 450 nm was recorded using an enzyme marker (800 TS, Bio-Tek, United States) after 2 h. The cell survival rate was calculated by the following formula:

$$Cell\;viability\;\left(\%\right)=\frac{As-Ab}{Ac-Ab}\times 100\%$$

where As is the absorbance of experimental wells (absorbance of wells containing cells, medium, CCK-8 solution and Mn-CDs/CDs solution); Ab is the absorbance of the blank wells (absorbance of the wells containing medium and CCK-8 solution); Ac is the absorbance of the control wells (absorbance of the wells containing cells, medium CCK-8 solution and no Mn-CDs/CDs solution).

Similarly, cell proliferation and cytotoxicity assay kits were used to verify the phototoxicity of Mn-CDs. OSCC-9 cells (5.0 × 103 cells per well) were inoculated in 96-well plates and co-cultured for 24 h. After adding Mn-CDs (100 μg/mL) or CDs (100 μg/mL) and continuing co-culture for 4 h. After the addition of a culture medium with H_2_O_2_ (50 μM, pH = 6.5), the OSCC-9 cells were irradiated using a 635 nm laser (300 mW·cm-2) irradiation for 5 min. After 20 h, the old medium was replaced with CCK-8 reagent and cell survival was calculated by recording the absorbance of each well at 450 nm using an enzyme marker (800 TS, Bio-Tek, United States). The cells incubated with the Mn-CDs or CDs without the addition of acidic H_2_O_2_ were irradiated by a 635 nm laser (300 mW·cm-2, 5 min) as controls.

To further verify the photodynamic efficiency of Mn-CDs and CDs, OSCC-9 cells (2.5 × 104 cells per well) were inoculated in 24-well plates and incubated for 24 h. Mn-CDs or CDs (100 μg/mL) were added for 4 h H_2_O_2_ (50 μM, pH = 6.5) was added, followed by irradiation under a 635 nm laser (300 mW·cm-2) for 5 min and co-staining with Calcein AM and PI after 20 h. Finally, imaging was performed by a Leica SP8 LSCM. Similarly, the group without H_2_O_2_ addition was used as a control.

#### 2.5.4 Cell apoptosis assay

Flow cytometry was conducted for cell apoptosis analysis. OSCC-9 cells were inoculated (2.5 × 104 cells per well, *n* = 4) in 24-well plates and incubated with Mn-CDs (100 μg/mL, 500 μL) or CDs (100 μg/mL, 500 μL) for 4 h. The H_2_O_2_ (50 μM, pH = 6.5) was added and then irradiated for 5 min using a 635 nm laser (300 mW·cm-2). Then, the Annexin V-FITC/PI Apoptosis Detection Kit was used for cell staining according to the protocol provided by the manufacturer. Apoptosis flow cytometric analysis was conducted by a flow cytometer (Dx FLEX, Beckman coulter, United States). The group without H_2_O_2_ addition was used as a control.

#### 2.5.5 Toxicological analysis

SD rats (male, ∼6 weeks old) were randomly divided into three groups (*n* = 3) and injected with the following drugs via tail vein: 1) PBS injection group; 2) Mn-CDs (100 μg/mL, 1 mL) injection group; 3) CDs (100 μg/mL, 1 mL) injection group. 7 days later, the rats were executed, and the heart, liver, spleen, lungs, and kidneys were collected. The main organs were fixed in 4% formalin overnight, embedded in paraffin, stained with hematoxylin and eosin (H&E), and observed with the optical microscope. White blood cells (WBC), red blood cells (RBC), hemoglobin (HGB), hematocrit (HCT), mean corpuscular volume (MCV), mean corpuscular hemoglobin (MCH), mean corpuscular hemoglobin concentration (MCHC) and mean platelet volume (MPV) were measured by blood routine examination. Meanwhile, blood biochemical analysis was conducted to examine the blood levels of alanine aminotransaminase (ALT), alkaline phosphatase (ALP), aspartate aminotransferase (AST), urea nitrogen (UREA) and creatinine (CREA).

### 2.6 Statistical analysis

All quantified data were presented as mean ± standard deviation (SD). The experiment was performed three times without special explanations. One-way analysis of variance (ANOVA) and Tukey’s test were used to determine differences between groups. A value of *p* < 0.05 was considered statistically significant.

## 3 Results and discussion

### 3.1 Physicochemical characterization of Mn-CDs and CDs

**FIGURE 1:**
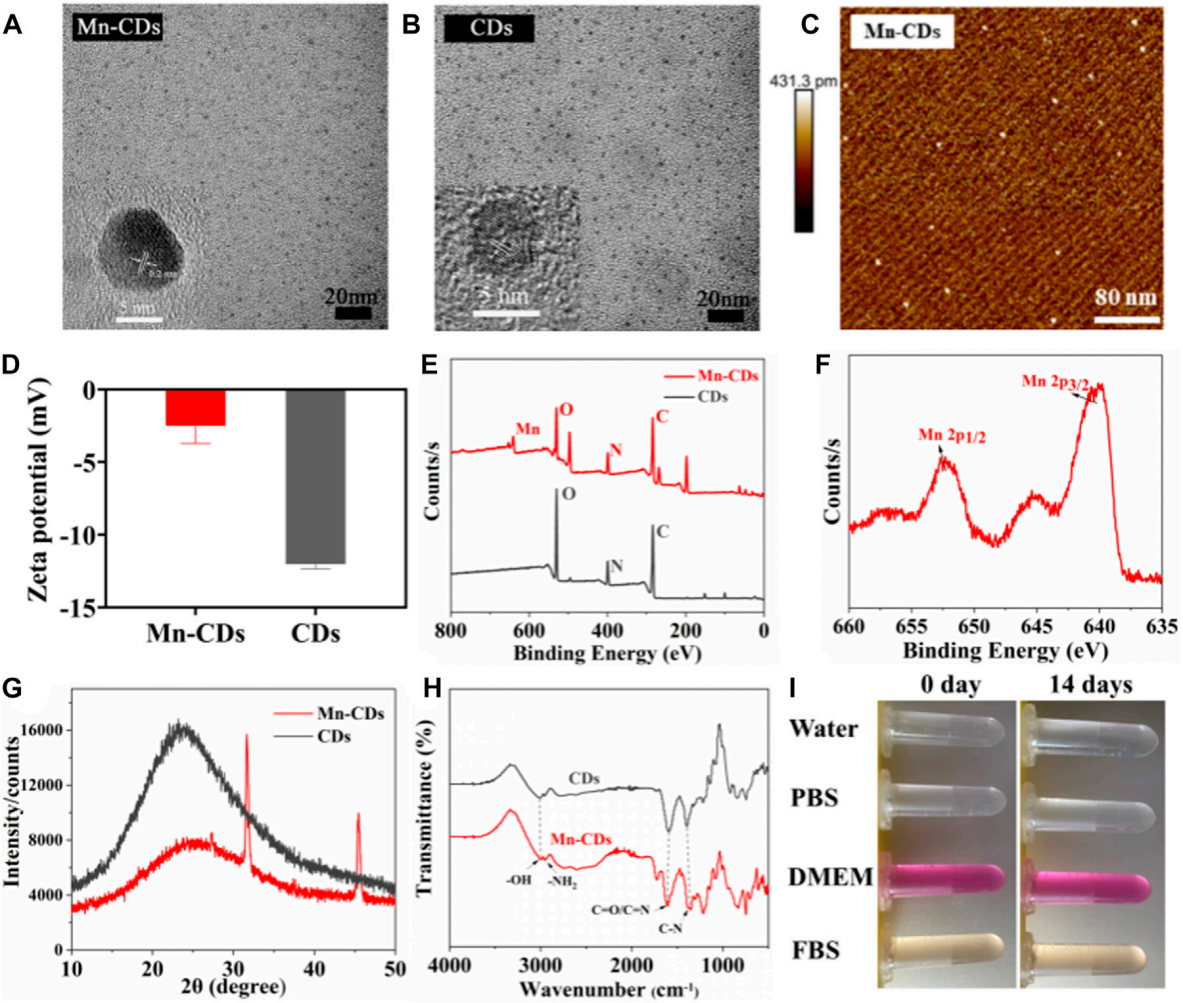
Physical and chemical characterization of Mn-CDs and CDs. **(A)** TEM of Mn-CDs. Inset: HR-TEM of Mn-CDs. **(B)** TEM of CDs. Inset: HR-TEM of CDs. **(C)** AFM of Mn-CDs. **(D)** The ζ potentials of the Mn-CDs and CDs in aqueous solution. **(E)** XPS spectra of Mn-CDs and CDs. **(F)** XPS spectra of Mn 2p for the Mn-CDs. **(G)** XRD of Mn-CDs and CDs. **(H)** FT-IR spectra of Mn-CDs and CDs. **(I)** The dispersed stability of Mn-CDs in different solutions.

The Mn-CDs were prepared using NaMnEDTA and OPD, as EDTA has a flexible structure and is a common starting material for CDs, and EDTA chelates metal ions to form saturated Schiff base-like structures (Wu et al., 2015). As shown by the TEM results (Figure 1A), the Mn-CDs exhibited a single uniformly distributed spherical morphology with an average diameter size of 2.7 nm (Supplementary Figure S1A). Meanwhile, the original CDs were synthesized by the same method using EDTA as the precursor, and their morphology was similar to that of Mn-CDs (Figure 1B) with an average particle size of approximately 3.0 nm (Supplementary Figure S1B). The HR-TEM images exhibited that Mn-CDs with an interlayer spacing of 0.2 nm have high crystallinity, which is consistent with the 0.21 nm lattice spacing of graphene (inset of Figure 1A), indicating that the Mn-CDs contain graphite-like structure (Jia et al., 2018a; Tian et al., 2021). Pristine CDs also displayed a similar morphology to that of Mn-CDs, which was confirmed by the 0.17 nm interlayer spacing in the CDs (inset of Figure 1B). Similarly, the AFM images showed that the average heights of Mn-CDs and CDs were about 0.34 nm and 0.41 nm, respectively, both of which were less than 1 nm, indicating that Mn-CDs and CDs were composed of a single layer of graphene. (Figure 1C; Supplementary Figure S2).

As shown in Figure 1D, the ζ potential value of Mn-CDs in water was −2.50 ± 0.98 mV, indicating the presence of carboxyl groups on surfaces (Sun et al., 2020). Notably, this value was more positive than that of Mn-free CDs (−12.04 ± 0.25 mV), possibly due to the existence of positively charged Mn2+ on the surface of Mn-CDs (Tian et al., 2021). This was confirmed by XPS. In the full-scan XPS spectrum, four characteristic absorption peaks related to C1s (284.06 eV, 53.79%), O1s (530.35 eV, 15.77%), N1s (398.96 eV, 11.55%), and Mn2p (640.40 eV, 2.12%) were recognized (Figure 1C). In vivid contrast, the CDs without Mn contained only three peaks: C1s (283.98 eV, 56.69%), O1s (530.16 eV, 23.48%), and N1s (399.21 eV, 12.56%). The XPS spectra of O1s and N1s of CDs and Mn-CDs show different quantities on species, suggesting that Mn doping induces changes in the local chemical environment of O and N elements of CDs (Liu et al., 2022). Moreover, the characteristic peak of Mn2p corresponding to MnO2 demonstrates that a considerable portion of manganese is doped into the CDs (Figure 1D) (Sun et al., 2019; Sun et al., 2021). In addition, a wide diffraction peak appears in the XRD pattern of the Mn-CDs at 25.5° (Figure 1G), with a slight peak shift at a larger angle (≈23.92° to ≈25.5°), compared to Mn-free CDs, suggesting that Mn doping on the carbon skeleton leads to a more disordered graphite-like structure (002) (Gao et al., 2018; Tian et al., 2021; Zhu et al., 2022).

FT-IR spectra confirmed the existence of carboxyl and amino groups embedded in the CDs with the observation of a sharp peak at 1,610 cm-1 corresponding to the stretching vibration of C=O and a broad peak at 3,000 cm-1 with respect to N–H absorption (Figure 1E), respectively. A manifestation of their zwitterionic characteristics, allows them to be well dispersed in aqueous solutions (Zhang et al., 2015; Zhu et al., 2022). In addition, the peak at 607 cm-1 corresponding to the Mn-O functional group and a new distinct peak at around 1,220 cm-1 caused by metal-ligand stretching vibrations furthermore indicates the existence of Mn (Liu et al., 2022; Dong et al., 2022). The results of ζ potential, XPS, XRD and FT-IR all demonstrate that Mn has been successfully doped into CDs, while the presence of a large number of hydrophilic functional groups on the surface of Mn-CDs improves the biocompatibility of the material itself.

As illustrated in Figure 1I and Supplementary Figure S3, the Mn-CDs and CDs were stable in different media (water, PBS, DMEM, and FBS) without any noticeable aggregation for 14 days at both ambient conditions and 4°C. Moreover, the hydrated particle size results further verified that the particle size of Mn-CDs is almost constant for up to 14 days (Supplementary Figure S4). All the above results indicated that the prepared Mn-CDs were highly stable.

**FIGURE 2:**
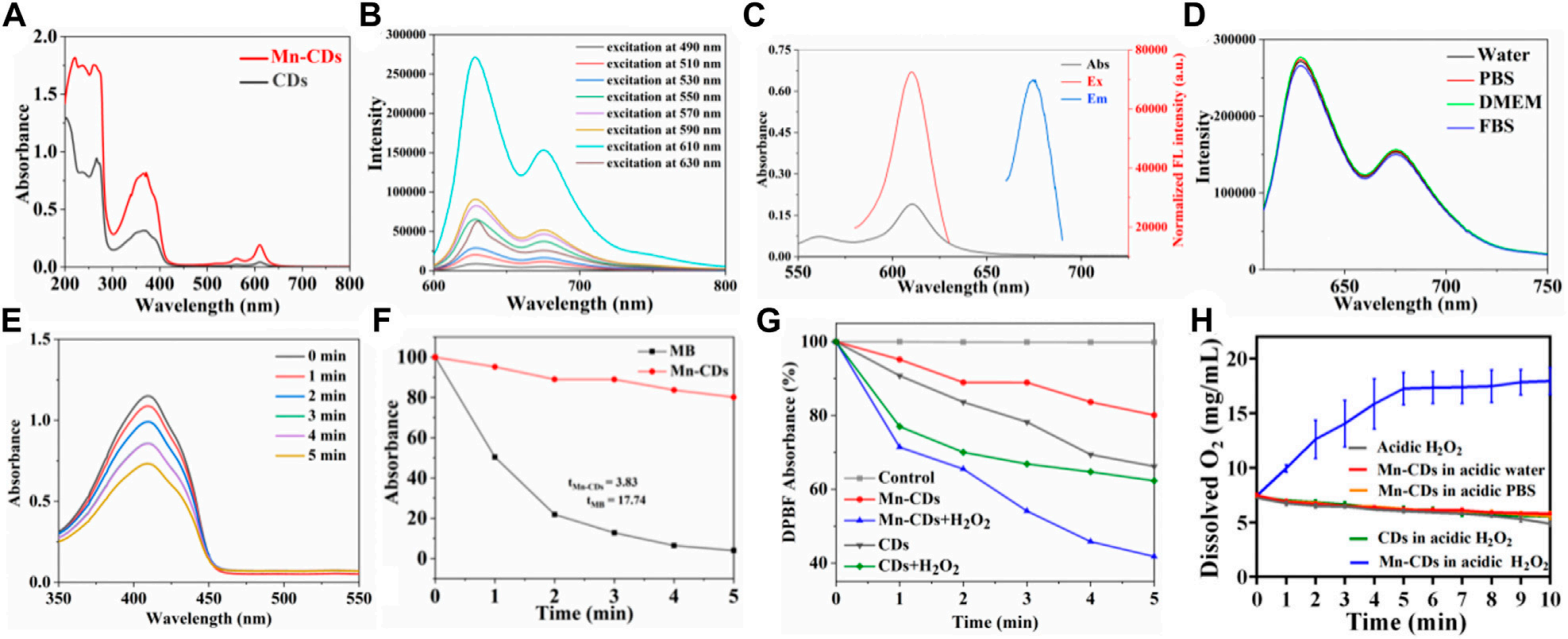
The photoactive characteristics and stability of Mn-CDs and CDs. **(A)** UV–VIS spectrum Mn-CDs and CDs in aqueous solution. **(B)** The FL of Mn-CDs **(C)** The absorption spectrum, excitation, and emission spectra of Mn-CDs in aqueous solution. **(D)** PL spectra of Mn-CDs in different media. **(E)** Absorption spectra of DPBF mixed with the Mn-CDs under 635 nm laser irradiation. **(F)** Decay curves of the DPBF absorption at 412 nm as a function of irradiation time. **(G)** Generation of 1O2 from Mn-CDs and CDs determined by a change in DPBF absorbance in the presence or absence of H_2_O_2_. **(H)** Dissolved oxygen concentrations of Mn-CDs and CDs in different reaction systems. Data are expressed as means ± s **(D)** (*n* = 3).

The UV–Vis and photoluminescence (PL) spectroscopy of the Mn-CDs and CDs solution are exhibited in Figure 2. The UV–Vis absorption spectra of Mn-CDs and CDs have multiple absorption peaks at ∼265 nm, 350–450 nm and 550–750 nm (Figure 2A), corresponding to π→π* jumps for aromatic C=C bonds and π→π* and n→π* jumps for aromatic π systems containing C=O, C=N and C=S bonds, respectively (Zhang et al., 2015; Pan et al., 2016). Moreover, the absorption intensity of Mn-CDs among 500–650 nm was stronger than that of CDs in the same conditions (Figure 2A), which was due to the metal-to-graphite charge-transfer transitions, revealing the chelation of Mn with N inside Mn-CDs (Tian et al., 2021). The fluorescence of Mn-CDs/CDs in aqueous solution was further investigated by PL spectroscopy at multiple excitation wavelengths (Figure 2B and Supporting Material; Supplementary Figure S5A). Like many other red-emissive CDs (Chen et al., 2021; Tian et al., 2021), the fluorescence emission of Mn-CDs is independent of the excitation wavelength, and the emission wavelength is 675 nm when the excitation wavelength is tuned from 490 to 630 nm (Figure 2C). This complex emission behavior is due to the surface state affecting the band gap of Mn-CDs. The surface state was similar to the molecular state, while the size effect was a result of the quantum dimension (Zhu et al., 2013). The fluorescence intensity (675 nm) was the most efficient when the excitation wavelength was 610 nm (Figure 2C). Compared to previous water-soluble Mn-doped CDs, their emission wavelengths are usually concentrated between 300 nm and 600 nm, mostly in blue-emissive and orange-emissive CDs, with excitation-dependent fluorescence properties (Irmania et al., 2020; Sun et al., 2021). UV excitation causes severe photodamage to biological tissues due to the low penetration of the emitted light, which limits the application of fluorescent biosensing (Zhu et al., 2022). Here, the as-prepared red-emissive Mn-CDs have better imaging contrast and can be directly used for intracellular imaging (Zhu et al., 2022). Similarly, the optimal emission wavelength and emission wavelength of CDs were 610 nm and 675 nm, respectively (Supplementary Figure S5B), and the doping of Mn does not cause any change in the fluorescence properties of CDs (Chen et al., 2021). Furthermore, the PL spectra of Mn-CDs in different physiological media (water, PBS, DMEM, and FBS) were investigated (Figure 2D). The results showed that the changes in excitation and emission wavelengths of Mn-CDs caused by different media were negligible, confirming that Mn-CDs were highly stable in different media, which is consistent with the stability results.

### 3.2 Photodynamic efficiency of Mn-CDs and CDs *in vitro*

To evaluate the capacity of Mn-CDs to produce 1O_2_, DPBF was used as the detection reagent because 1O_2_ could cause an irreversible decrease in the absorbance of DPBF at 412 nm (Zhang et al., 2017). The absorption of DPBF decreases with irradiation time in the presence of Mn-CDs and MB under 635 nm laser irradiation (300 mW·cm-2) (Figure 2E; Supplementary Figure S6). Based on the decay curves of the DPBF absorption (Figure 2F), the 1O_2_ quantum yield of Mn-CDs was derived as ≈2.27.

Previous studies have shown that a series of carbon nanomaterials possess inherent peroxidase activity (Sun et al., 2015; Liu et al., 2023). CDs possess high enzymatic activity due to their small size, good biocompatibility, and tunable surface. Notably, several mono- and polymetallic doping of CDs exhibited better peroxide-mimetic enzyme activity due to the metal’s valency change and enhanced electron transfer (electron giving and accepting) ability (Jia et al., 2018a; Chen et al., 2021; Liu et al., 2022; Xing et al., 2022). Here, based on the presence of Mn in the carbon dots confirmed by ζ potential, XPS, XRD detection, and FT-IR spectra, we speculated that the Mn-CDs have good peroxidase activity and could catalyse H_2_O_2_ to produce O2 and enhance PDT efficiency. Next experiments were performed to verify whether Mn-CDs could enhance PDT efficacy. As shown in Figure 2E, Mn-CDs produced significantly more 1O_2_ than CDs groups in a weak acid and rich H_2_O_2_ environment, with a decrease in absorbance of DPBF at 412 nm decreased by approximately 58.23% and 37.69%, respectively. This may be because Mn^2+^ can react with H_2_O_2_ to form MnO_2_, and O_2_ was generated by MnO_2_ in the presence of H_2_O_2_ and H+ as the following reactions:

$${Mn}^{2+}+H_{2}O_{2}\;\to \;{2H}^{+}+{MnO}_{2}$$ (2)

$${MnO}_{2}+H_{2}O_{2}+{2H}^{+}\to \;{Mn}^{2+}+{2H}_{2}O+O_{2}↑$$ (3)

Indeed, the presence of dissolved oxygen from the mixture of Mn-CDs and acidic H_2_O_2_ could be detected with an oxygen probe, whereas no oxygen was produced in the absence of Mn-CDs and acidic H_2_O_2_ (Figure 2H), which is in agreement with the previous study (Jia et al., 2018a).

Therefore, we anticipated that the PDT efficiency of Mn-CDs would be distinctly improved by the oxygen-burst strategy in TME.

### 3.3 Cellular uptake

**FIGURE 3:**
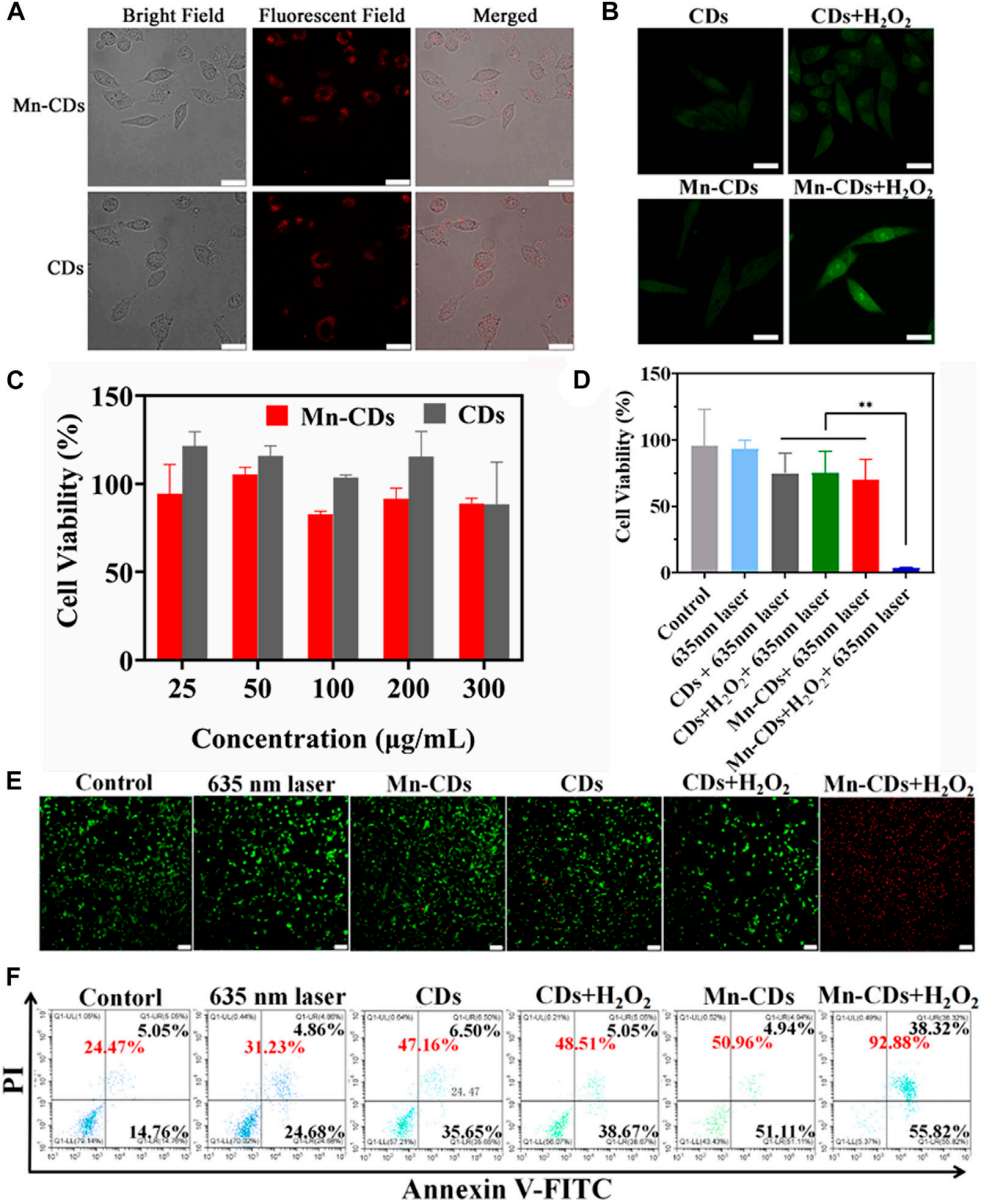
*In vitro* PDT efficiency of Mn-CDs and CDs. **(A)** Cellular uptake of Mn-CDs and CDs (Scale bar: 10 μm). **(B)** FL images of SOSG-stained OSCC-9 cells (Scale bar: 10 μm). **(C)** Cell viability of OSCC-9 cells incubated with Mn-CDs and CDs with different concentrations under the dark. **(D)** Cell viability of OSCC-9 cells treated with Mn-CDs and CDs with or without H2O2 under 635 nm laser irradiation (300 mW·cm^−2^). **(E)** The images of OSCC-9 cells co-stained by Calcein AM and PI after different treatments (Scale bar: 100 μm). **(F)** Flow cytometry analysis of apoptosis on OSCC-9 cells after co-incubation with various formulations.

Based on the excellent PL performance of Mn-CDs and CDs, we performed cellular uptake studies using LSCM. As shown in Figure 3A, after co-culture of Mn-CDs/CDs with OSCC-9 cells, red fluorescence of the cytoplasm was observed, indicating that Mn-CDs/CDs were able to effectively enter the cells and localise in the cytoplasm (Jia et al., 2018a; Tian et al., 2021).

### 3.4 *In vitro* PDT efficacy of Mn-CDs and CDs

We next investigated the production of 1O2 during intracellular PDT. SOSG reagent was highly selective for 1O2 and emitted green fluorescence like that of fluorescein in the presence of 1O2. As shown in Figure 3B, OSCC-9 cells treated with Mn-CDs and CDs produced negligible intracellular 1O2 signals (green) after irradiation with a 635 nm laser (300 mW·cm-2, 5 min). This may be due to insufficient oxygen supply. As expected, the green fluorescence signal was strengthened in the Mn-CDs group upon acidic H_2_O_2_, whereas no significant changes were seen in the CDs group. This suggests that Mn^2+^ reacts with H_2_O_2_ to produce O_2_, which favors potentially enhanced cytotoxic properties of Mn-CDs against OSCC-9 cells.

Encouraged by the remarkable cellular uptake of Mn-CDs and CDs in OSCC-9, we explored their cytotoxicity *in vitro* with/without 635 nm laser irradiation to demonstrate their anti-tumor efficiency. Under dark conditions, the cytotoxicity of Mn-CDs and CDs was negligible even as the concentration increased to 300 μg/mL (Figure 3C), confirming their good biocompatibility and low side effects. In addition, the *in vitro* anti-tumor efficiency of Mn-CDs was investigated by simulating micro-acidic and H_2_O_2_-rich TME (Figure 3D). After being irradiated with a 635 nm laser (300 mW·cm-2, 5 min), the cell viability of Mn-CDs and CDs exhibited moderate phototoxicity (74.85% and 70.16%) to OSCC-9 cells with the concentration of 100 μg/mL, which may be due to the insufficient oxygen supply (Jia et al., 2018a; Chen et al., 2018). On the contrary, the cell viability of the Mn-CDs with the addition of acidic H_2_O_2_ disastrously decreased to 3.58%, while CDs + acidic H_2_O_2_ group did not decrease significantly. Therefore, PDT with the enhanced oxygen production triggered by Mn-CDs manifests a more excellent anti-tumor effect than the single CDs.

To more visually confirm the cytotoxicities of Mn-CDs, LIVE/DEAD staining assay was completed using a double staining Calcein AM/PI (green fluorescence for living cells/red fluorescence for dead cells) (Figure 3E). Cells in the control and laser-irradiated groups showed green fluorescence, but the red fluorescence signal was undetectable, indicating the lack of killing effect of light irradiation. An increased red fluorescent signal was observed in the Mn-CDs and CDs groups compared to negligible apoptosis in the control group, which was similar to the results of cell cytotoxicity. Furthermore, cell death was most pronounced in the group of Mn-CDs with added H_2_O_2_, which demonstrated that Mn^2+^ could significantly improve the efficiency of PDT. In contrast, no significant change in red fluorescence signal in the group of CDs added with H_2_O_2_ was due to the inability of these Mn-free CDs to generate O_2_. Similar results were obtained in the flow cytometry assay (Figure 3F). As expected, the Mn-CDs group showed the highest levels of cell apoptosis rates (92.88%) because of the continuous oxygen supply via CAT-like activity of Mn^2+^ in acidic H2O2 (Supplementary Figure S7). These results clearly indicate that Mn-CDs can be used as a potentially efficient PDT photosensitizer in hypoxic, slightly acidic and H_2_O_2_-rich TEM.

### 3.5 Biocompatibility of Mn-CDs and CDs *in vivo*

**FIGURE 4:**
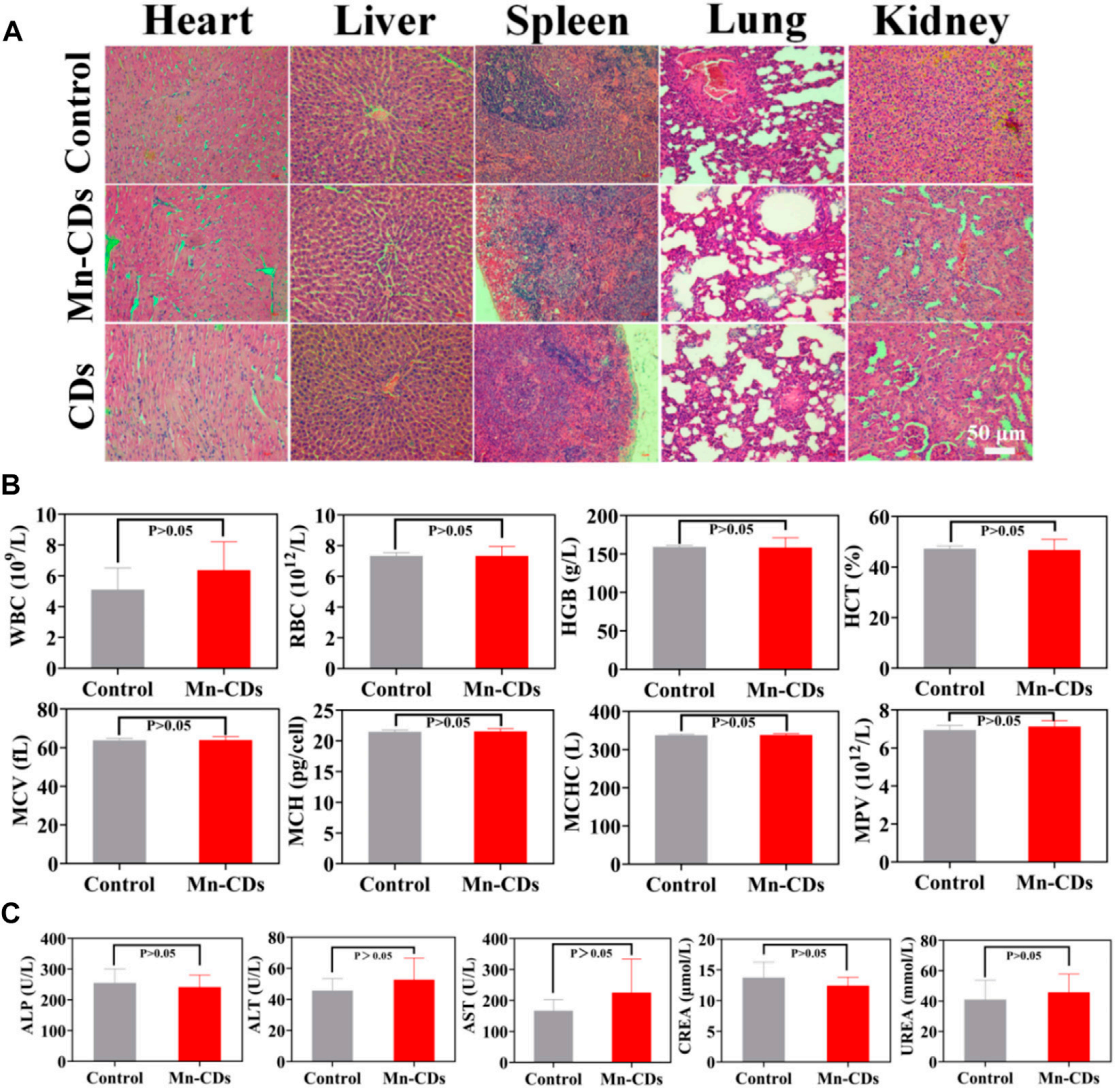
*In vivo* biosafety of Mn-CDs and CDs. **(A)** H&E staining of major tissues from SD rats after different treatments. **(B)** Blood routine examination of rats treated with PBS and Mn-CDs (10 mgkg^−1^). **(C)** Blood biochemical analysis of ALT, AST, ALP, UREA and CREA after injection of PBS and Mn-CDs (10 mgkg^−1^) for 7 days (*n* = 3).

The biosafety of Mn-CDs and CDs was further investigated *in vivo* via different methods. The H&E staining of major organs like the heart, liver, spleen, lung, and kidney suggested that no tissue damage or inflammation of major organs was observed in the Mn-CDs treated group compared to the healthy mice group injected with PBS only for 7 days (Figure 4A), verifying the negligible side effects of the formulation. Similarly, CDs showed no significant toxic effects (Figure 4A).

Moreover, we also performed routine hematological studies, and serum biochemical analyses at the levels of ALT, AST, ALP, UREA, and CREA. When quantum dots are introduced into the body by intravenous injection, the first system with which they interact is the blood and blood components (Ji et al., 2014). Quantum dots and other nanoparticles are exogenous stimuli and as such can induce inflammatory responses, alter the activity of the immune system and affect associated blood factors such as white blood cell counts (Hauck et al., 2010). In addition, the toxicological profile of nanomaterials may be determined by the chemical composition, size, shape, aggregation and surface coating of the nanomaterials and may lead to changes in haematological indicators such as red blood cells or haemoglobin (Hauck et al., 2010). Therefore, the following standard haematological indicators were selected for analysis: WBC, RBC, HGB, HCT, MCV, MCH, MCHC, and MPV. Haematological measurements (Figure 4B; Supplementary Figure S8A) between Mn-CDs/CDs treated rats and control rats were not significantly different (*p* > 0.05) and showed no significant toxic effects. Once the nanoparticles have left the bloodstream and reached the liver and kidneys, the toxic effects of the nanoparticles can be assessed by measuring serum indicators of liver and kidney damage. Liver function, hepatocellular damage and reduced bile flow can be assessed by measuring various factors in serum (Hauck et al., 2010). Decreased liver function and reduced bile flow can be assessed by substances produced by the liver, including ALP, ALT, and AST, while indicators of kidney function usually include UREA and CREA (Choi et al., 2007; Hauck et al., 2010). The results of the serum biochemical analysis (Figure 4C; Supplementary Figure S8B) did not indicate any significant toxicity in the Mn-CDs/CDs group compared to the control group, further confirming the good biosafety of Mn-CDs.

These data collectively demonstrated the satisfactory biosafety of Mn-CDs and CDs without generating evident systemic toxicity and side effects.

## 4 Conclusion

In conclusion, we have first exploited the one-pot solvent heating method to successfully synthesize the hydrophilic Mn-CDs, which exhibited excellent peroxidase-like activity for generating O_2_ in the weak acid and rich H_2_O_2_ TME and 1O_2_ production capability under 635 nm laser (300 mW·cm-2). The *in vitro* anticancer experiments suggest that the combination of intrinsic peroxidase-like activity plus enhanced PDT efficacy endows Mn-CDs 92.88% apoptosis rate against OSCC-9 cells. More importantly, Mn-CDs with ultrasmall size showed good biocompatibility without causing substantial inflammatory or pathological damage to normal tissues. Overall, this work would provide a potential photosensitizer with high PDT efficiency for the treatment of oral squamous cell carcinoma and offers a new idea for subsequent research on the treatment of solid oral squamous cell carcinoma.

## Data Availability

The original contributions presented in the study are included in the article/Supplementary Material, further inquiries can be directed to the corresponding authors.
