# Supplementary material for: Hypoxia mitigation by manganese-doped carbon dots for synergistic photodynamic therapy of oral squamous cell carcinoma
Source: Front Bioeng Biotechnol. 2023 Apr 20;11:1153196. doi: 10.3389/fbioe.2023.1153196 (PMC10157228; doi:10.3389/fbioe.2023.1153196)
Supplement: Supplementary file 1 [file DataSheet1.docx]

**Supplementary Materials:**

**Hypoxia mitigation by manganese-doped carbon dots for synergistic photodynamic therapy of oral squamous cell carcinoma**

Zhe Zhang^1^, Yongzhi Xu^1^, Tingting Zhu^1^, Zhiqin Sang^1^, Xiaoli Guo^1^, Yu Sun^1^, Yuanping Hao^1,2*^, Wanchun Wang^1,2*^

1 School of Stomatology of Qingdao University, Qingdao 266003, China.

2 Qingdao Stomatological Hospital Affiliated to Qingdao University, Qingdao 266001, China.

*Correspondence: yphao@qdu.edu.cn (Y.H.); wangwanchun2019@qdu.edu.cn (W.W.);

Tel.: +86-166-2238-0102 (Y.H.); +86-138-5320-1386 (W.W.); Fax: +86-0532-82796465 (Y.H.)


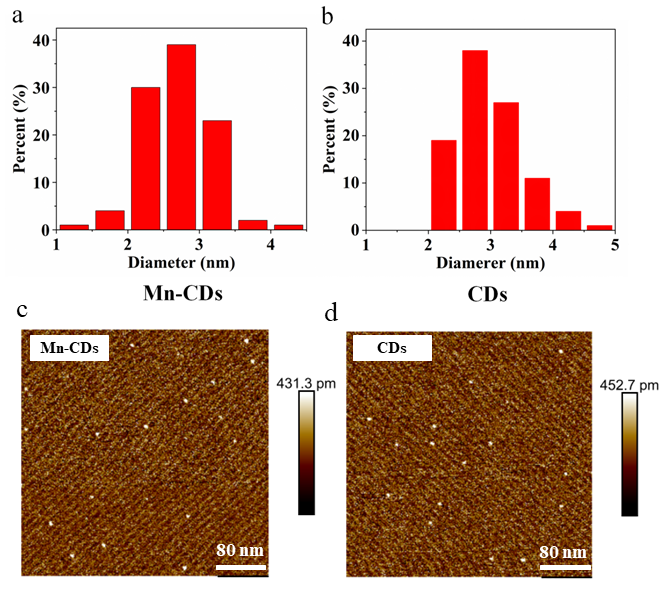


**Fig. S1. (**a) Particle size distribution of Mn-CDs. **(**b) Particle size distribution of CDs.


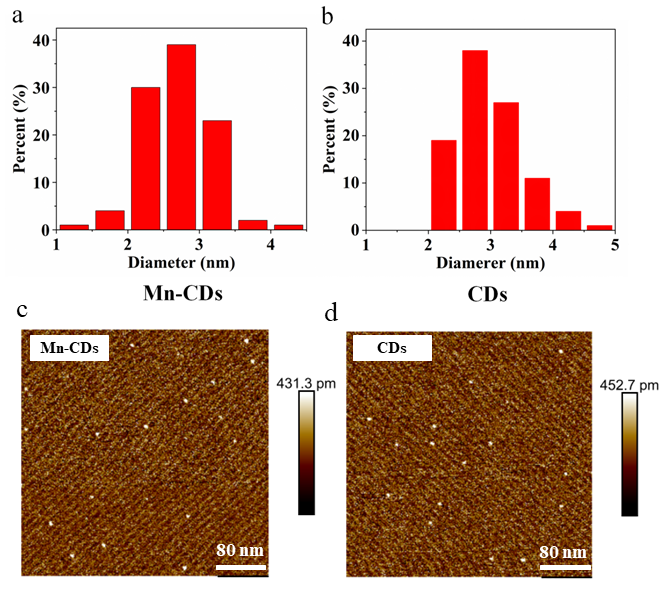


**Fig. S2.** AFM of CDs.

**
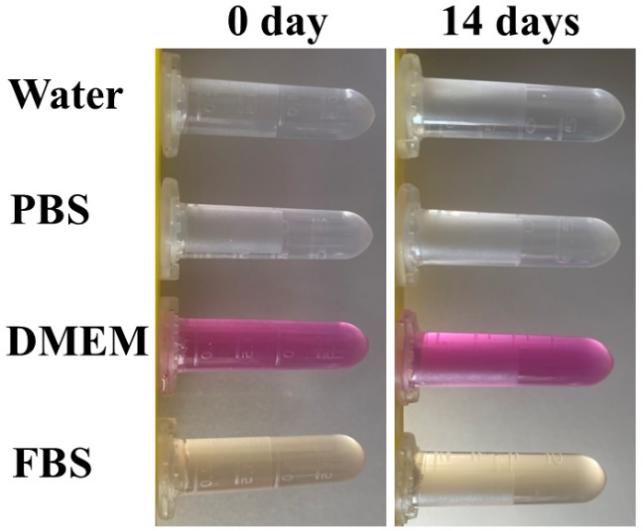
**

**Fig. S3.** The dispersed stability of the CDs in physiological mediums.


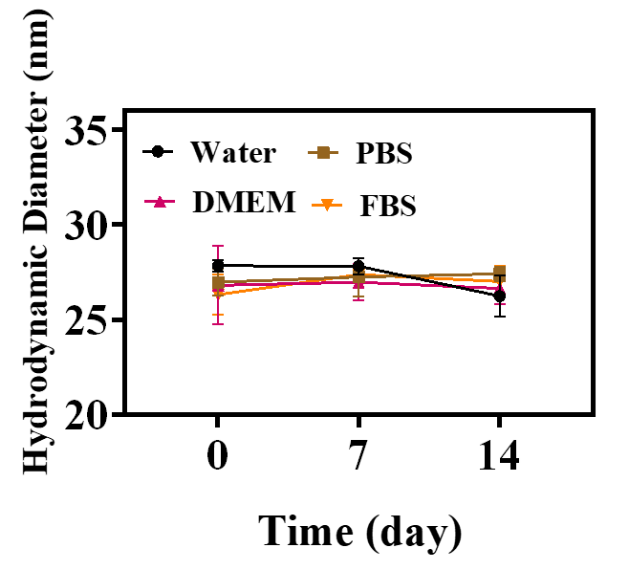


**Fig. S4.** The hydrated particle size of Mn-CD assembly in various solutions for 14 days.

**
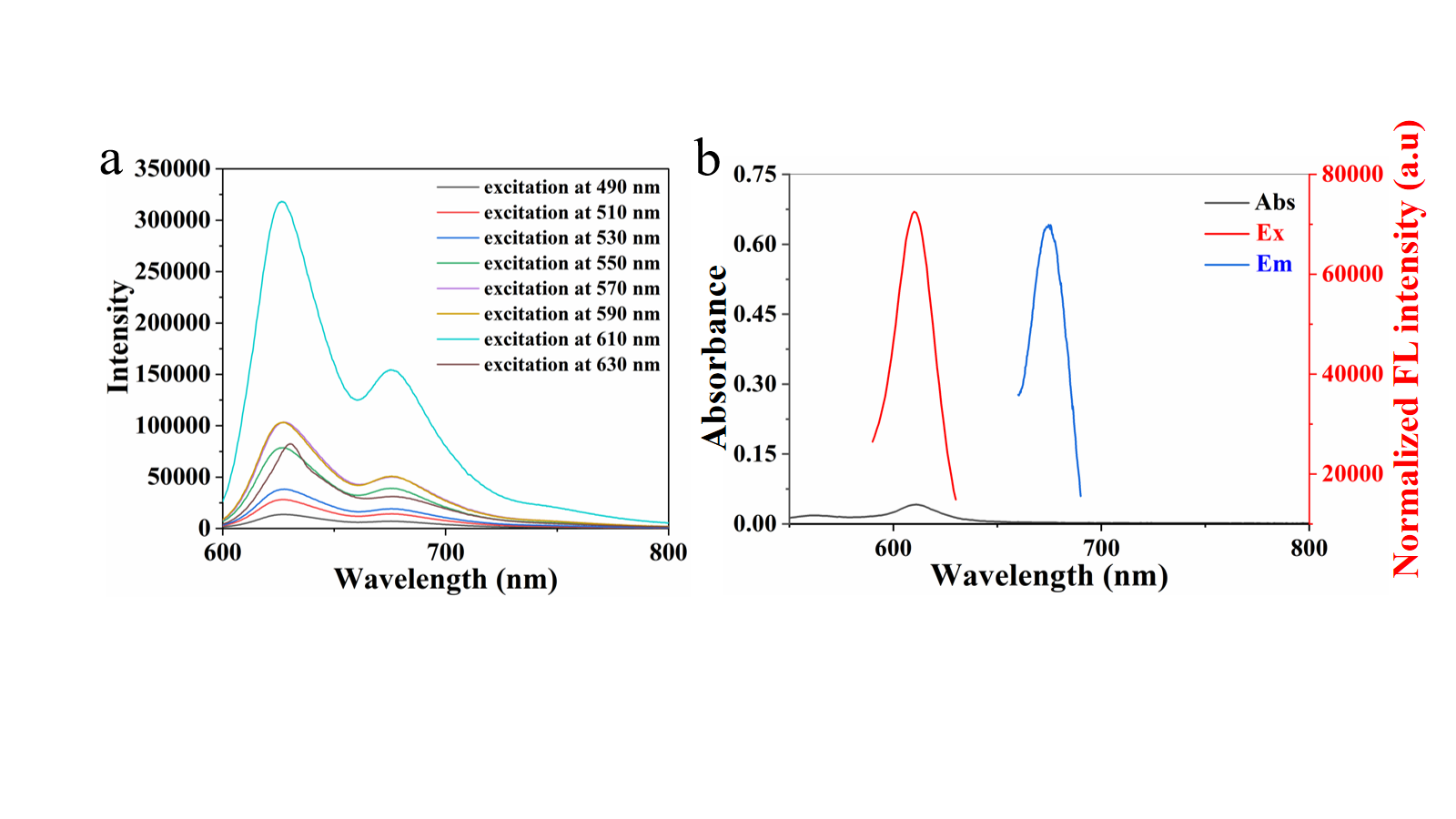
**

**Fig. S5.** (a) FL spectrum of CDs. (b) Absorption spectra, excitation spectrum, and emission spectrum of CDs in aqueous solution.


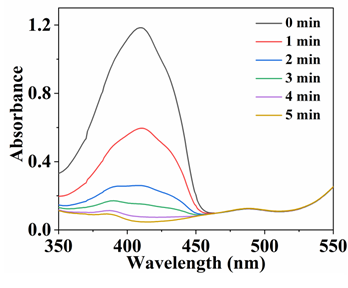


**Fig. S6.** Absorption spectra of DPBF mixed with the MB with 635 nm laser irradiation.

.
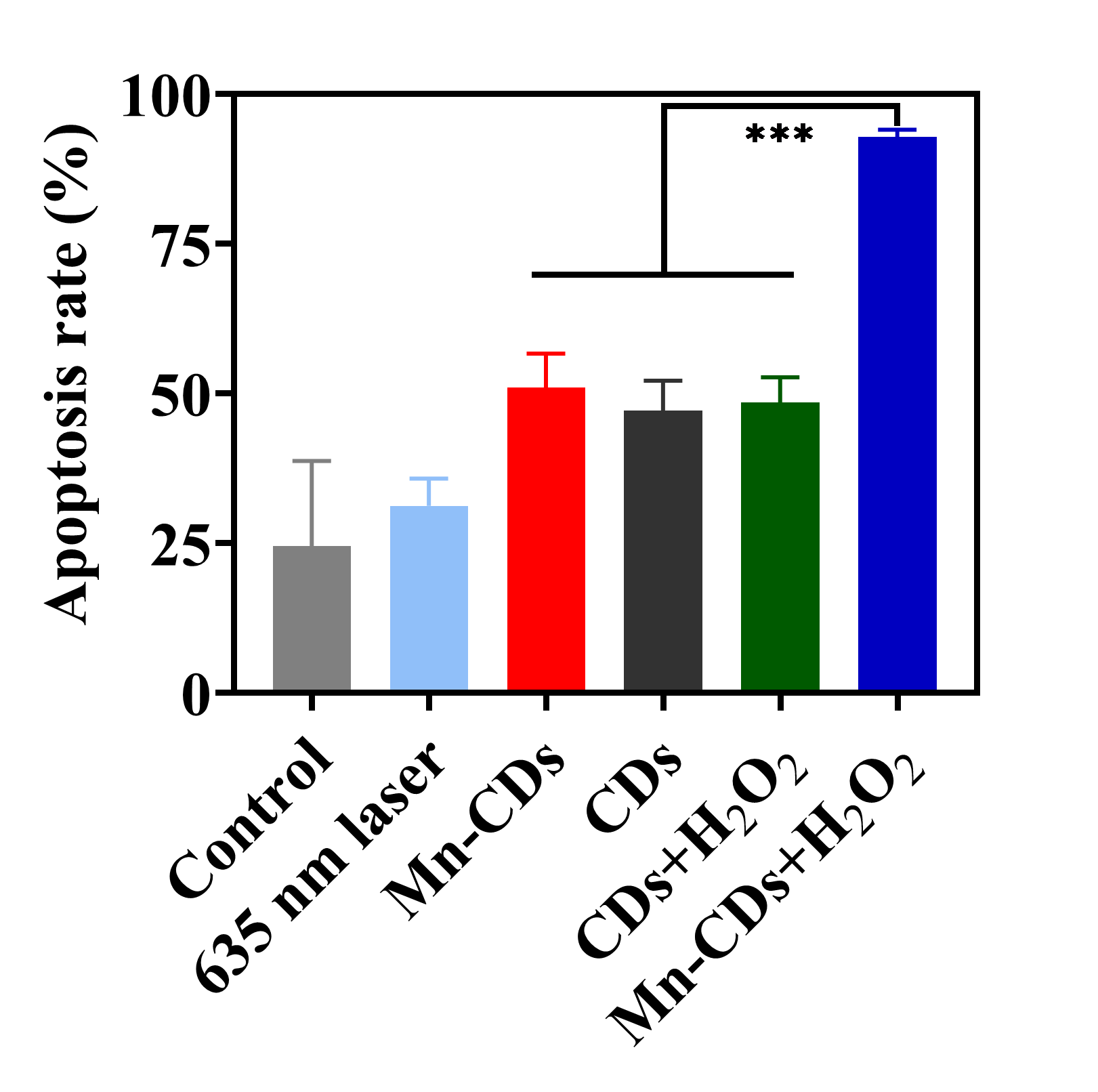


**Fig. S7.** Quantitative analysis of apoptosis after different treatments by flow cytometry. Data are expressed as means ± s. d. (n = 3).

**
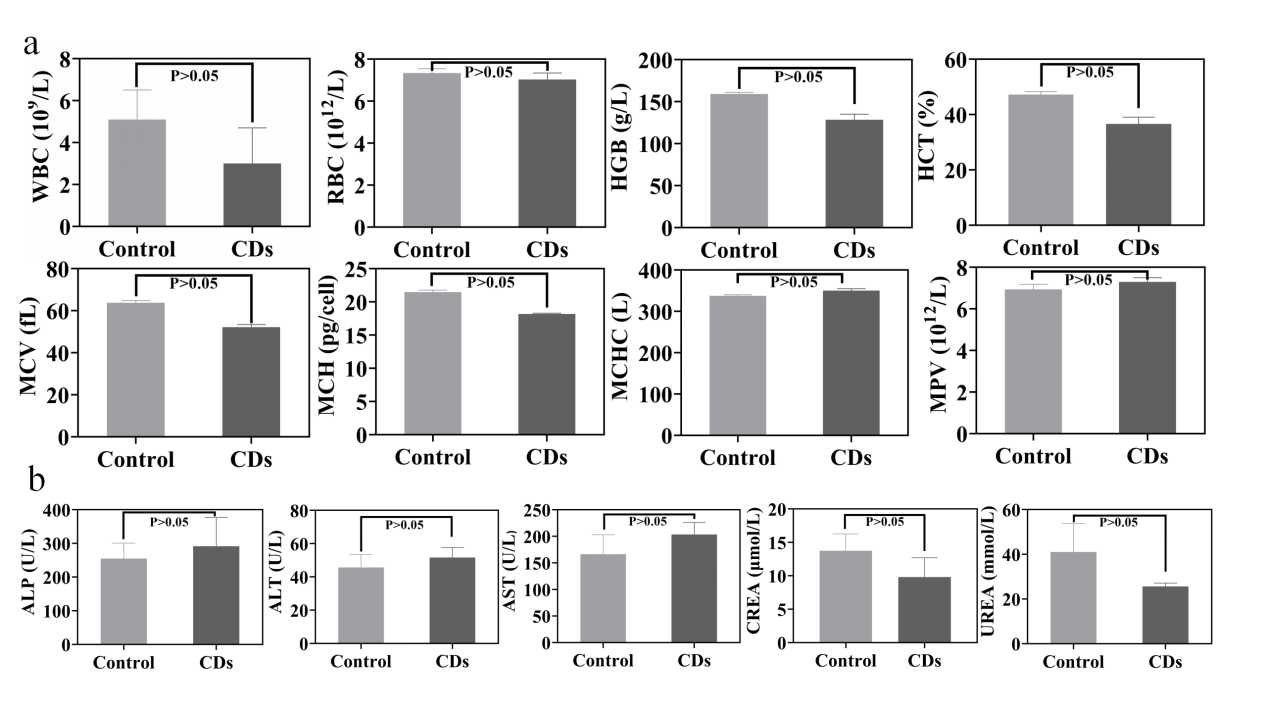
**

**Fig. S8.** (a) Blood routine examination of male SD rats treated with PBS and CDs (10 mg kg^−1^). After 7 days of injection, no significant difference in hematological data in all groups was found. The statistic was based on 3 rats per data point. (b) Measurement of serum biochemical parameters. After injection of PBS and CDs (10 mg kg^−1^) for 7 days, three indicators, ALT, AST and ALP for hepatic function, and UREA and CREA for renal function, were evaluated *via* blood samples from rats. The statistic was based on three rats per data point.
